# Larvicidal Activity of *Bunium persicum* Essential Oil and Extract against Malaria Vector, *Anopheles stephensi*

**Published:** 2018-03-18

**Authors:** Hassan Vatandoost, Arezoo Rustaie, Zeynab Talaeian, Mohammad Reza Abai, Fatemeh Moradkhani, Mahdi Vazirian, Abbas Hadjiakhoondi, Mohammad Reza Shams-Ardekani, Mahnaz Khanavi

**Affiliations:** 1 Department of Medical Entomology and Vector Control, School of Public Health, Tehran University of Medical Sciences, Tehran, Iran; 2 Department of Chemical Pollutants and Pesticides, Institute for Environmental Research, Tehran University of Medical Sciences, Tehran, Iran; 3 Department of Pharmacognosy, School of Pharmacy and Persian Medicine and Pharmacy Research Centre, Tehran University of Medical Sciences, Tehran, Iran; 4 Department of Traditional Pharmacy, School of Traditional Medicine, Tehran University of Medical Sciences, Tehran, Iran; 5 Faculty of Land and Food Systems, University of British Columbia, Vancouver, Canada

**Keywords:** *Anopheles stephensi*, *Bunium persicum*, Larvicidal activity, Extract, Essential oil

## Abstract

**Background:**

Malaria, a mosquito-transmitted disease, is still a major human health problem all over the world. Larviciding is a component of comprehensive control program to overcome the disease. Negative aspects of synthetic insecticides application, such as environmental safety concerns, have favored use of natural insecticides.

**Methods:**

Larvicidal activity of essential oil, extracts and fractions of a wild grown and a cultivated type of *Bunium persicum* fruits against malaria vector *Anopheles stephensi* was assessed according to the method described by WHO.

**Results:**

*Bunium persicum* showed remarkable potency against *An. stephensi* larvae. LC _50_ values for essential oil, total extract, petroleum ether fraction and methanol fraction were 27.4284, 64.9933, 85.9933 and 255.7486ppm for wild type, and 21.3823, 63.2580, 62.7814 and 152.6357ppm for cultivated one.

**Conclusion:**

The results of this study suggest *B. persicum* as a valuable source of natural insecticides against malaria vector *Anopheles stephensi*.

## Introduction

Despite progresses made over the past decades to decline the mortality rate of malaria all over the world, it is still prevalent in some tropical countries and areas with about 200 million affected cases in 2013. Vector control interventions have had substantial contribution on the recent reduction in global malaria burden. Larviciding, with the aim of adult vector density reduction, as an auxiliary to core interventions, is helpful especially in urban regions, where breeding of vectors take places in permanent or semi-permanent aquatic habitats (1). The mosquito *Anopheles stephensi* is one of the six main vectors of human malaria in southern parts of Iran (2). Larvicidal potentials of some herbal extracts and essential oils on *An. stephensi* larvae have been investigated previously (3–5).

*Bunium persicum* is a perennial plant belonging to Apiaceae family, growing wild in Iran (6). The fruit of *B. persicum* is used as spice, antiseptic and carminative agent (7). Several studies have analyzed essential oil composition of the fruits and mostly reported γ-terpinene, cuminaldehyde and ρ-cymene as main components (8–10). Kaempferol, caffeic and pcoumaric acid have been isolated from polar fraction of the fruits as major antioxidant constituents (11) but according to our knowledge no other comprehensive study has been organized to identify other phytochemicals in the extract. Overexploitation and unscientific harvesting of *B. persicum* as well as climate changes, has threatened its existence in wild (12). Cultivation of endangered species could preserve their genetic resources (13). In recent years, *B. persicum* is cultivated in limited areas in Iran especially in Khorasan Razavi Province. As a part of our ongoing studies on larvicidal activity of plants extracts and essential oils against *An. stephensi* (4, 5, 14–20), in the present study, we have studied larvicidal activity of the essential oil, extract and fractions from *B. persicum* fruits against late third instar larvae of *An. stephensi*. Moreover, we have compared the activities of a wild and a cultivated type.

## Materials and Methods

### Plant material

The fruit of wild *B. persicum* was purchased from Kerman, and cultivated type was supplied from agricultural research fields of Ferdowsi University of Mashhad (2013). The samples were authenticated at the Herbarium of Faculty of Pharmacy, Tehran University of Medical Sciences, where voucher specimens were deposited (PMP-649 and PMP-689).

### Essential oil preparation

100g powdered fruits of cultivated and wild *B. persicum* were subjected to hydrodistillation for 3 hours using Clevenger type apparatus. The obtained essential oils were dried over anhydrous sodium sulfate and kept in refrigerator until needed.

### Extraction and fractionation

250g dried and powdered fruits from both samples were separately extracted with meth anol (5× 1.5L) to afford total methanol extracts. The solvent was removed under reduced pressure by rotary evaporator at 40 °C, and subsequently lyophilized by freeze dryer at −40 °C for 24h (Lyotrap Ultra, LTE Scientific Ltd., Oldham, UK). Fractionation of total extracts was performed with sufficient volumes of petroleum ether, ethyl acetate and methanol. The fractions were then concentrated to dryness by rotary evaporation.

### Larval mortality bioassay

*Anopheles stephensi* larvae (Bandar Abbas strain) were supplied by the Department of Medical Entomology, Tehran University of Medical Sciences. The mosquito colony was maintained under a constant insectarium condition at 27 °C and 75–85% relative humidity with 12:12 light and dark photoperiod. Late third and early fourth instars larvae were used for experiments.

Larvicidal activity of total extracts, fractions and essential oils were evaluated according to the procedure recommended by WHO (21). The larvae were exposed to different concentrations of samples for 24 hours. Tests were carried out in four replicates. One ml of solvents (DMSO for essential oil and petroleum ether fraction, DMSO2: water 3 for total extract and ethanol for methanol fraction) were added separately into control bakers. Mortality was scored 24 hours post exposure.

### Analysis method

The mortality percentages were calculated and corrected relative to the associated controls using Abbott’s formula (22). The concentration-mortality data were subjected to Probit analysis (23) and lethal concentrations (LC _50_ and LC _90 _) were determined with 95% confidence intervals from the regression lines.

## Results

**Fig. 1.**
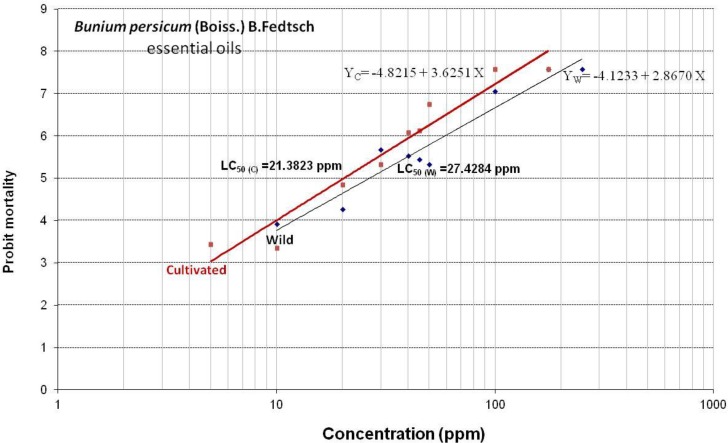
Comparison of lethal concentrations (LC _50 _) of cultivated and wild types of *Bunium persicum* essential oils against larvae of *Anopheles stephensi*

**Fig. 2.**
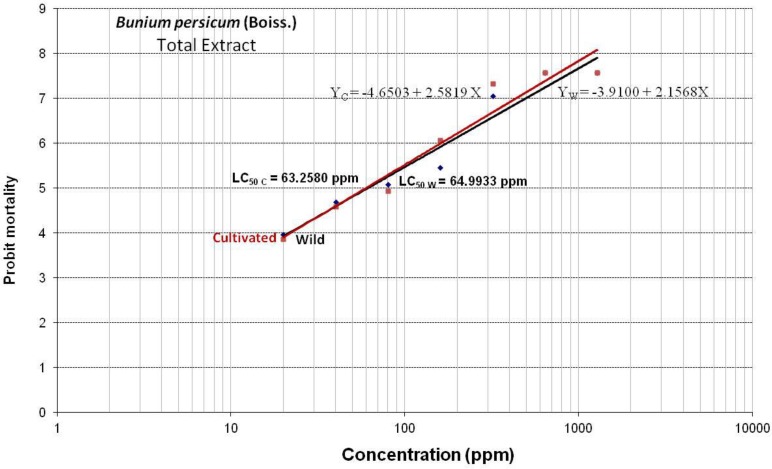
Comparison of lethal concentrations (LC _50 _) of cultivated and wild types of *Bunium persicum* total extracts against against larvae of *Anopheles stephensi*

**Fig. 3.**
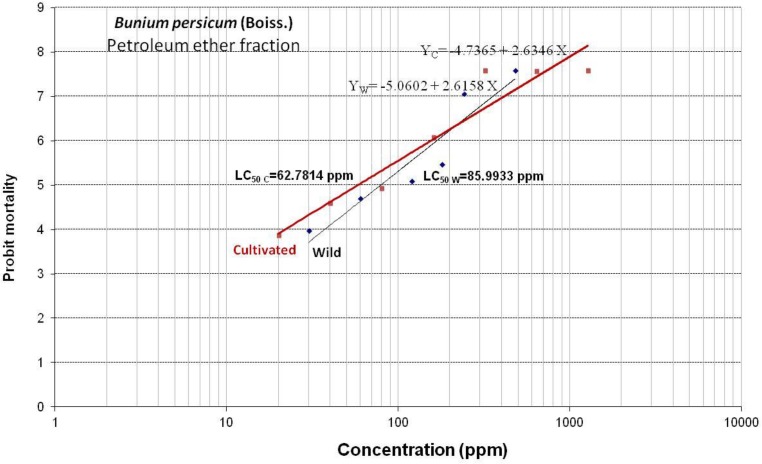
Comparison of lethal concentrations (LC _50 _) of cultivated and wild types of *Bunium persicum* petroleum ether fraction against against larvae of *Anopheles stephensi*

**Fig. 4.**
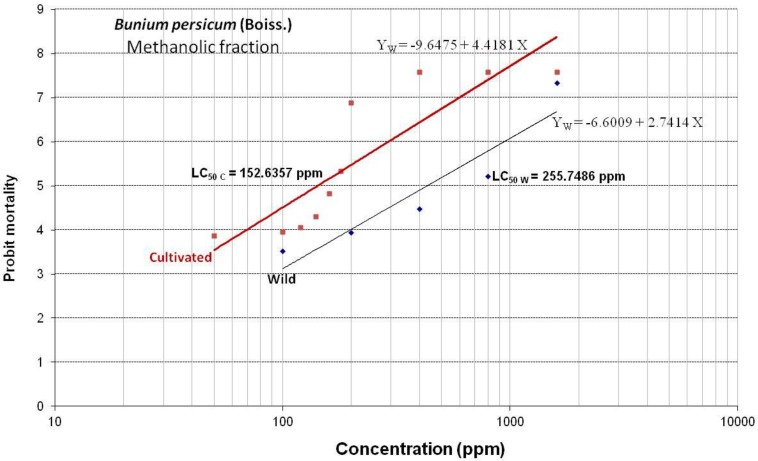
Comparison of lethal concentrations (LC _50 _) of cultivated and wild types of *Bunium persicum* methanol fraction against larvae of *Anopheles stephensi*

Hydro distillation of wild and cultivated *B. persicum* fruits yielded 2.5% and 2.25% (w/w) essential oil respectively. Both essential oils had a lot of commonalities in composition. γ-Terpinene (30.77% and 27.57%), cuminaldehyde (20.49% and 21.1%), ρ-cymene (20.1% and 18.32%) and γ-terpinen-7-al (8.29% and 7.84%) constituted main components in the wild and cultivated oils respectively (24). The results of larvicidal activity of essential oils, total extracts, petroleum ether and methanol extracts against *An. stephensi* under insectary condition are presented in table 1 and plotted in Figs. 1 to 4. All tested samples showed significant anti-larval effect against the malaria vector *An. stephensi*, of which, the essential oils from cultivated and wild types with LC _50_ values of 21.3823ppm and 27.4284ppm were the strongest samples and methanol fractions with LC _50_ values of 152.6357ppm and 255.7486 ppm exhibited least larvicidal activity among the samples. Comparison of lethal concentration values of efficient tested samples reveals there is no difference in efficacy of them between wild and cultivated types.

**Table 1.** Probit regression line parameters of essential oil, total extract, petroleum ether and methanol fraction of wild and cultivated *Bunium persicum* fruits against *Anopheles stephensi*

**Specimen**	**Intercept**	**Slope ± SE**	**LC_50_**	**95% CI**	**LC_90_**	**95% CI**	**χ<sup>2</sup>**	**P**
**W EO**	−4.1233	2.8670 ± 0.471	27.4284	19.7868-35.0421	76.7752	56.3038-139.1275	41.682	<0.05
**C EO**	−4.8215	3.6251 ± 0.486	21.3823	13.6913-25.9482	48.2608	38.6334-68.5159	33.107	<0.05
**W T**	−3.9100	2.1568 ± 0.270	64.9933	44.8917-89.5814	255.3195	170.9457-498.5673	16.725	<0.05
**C T**	−4.6503	2.5819 ± 0.181	63.2580	55.8062-71.3261	198.3795	167.9464-243.6008	10.718	<0.05
**W PE**	−5.0602	2.6158 ± 0.502	85.9933	47.2631-130.9756	265.7116	166.2611-869.8822	28.442	<0.05
**C PE**	−4.7365	2.6346 ± 0.186	62.7814	55.4789-70.6893	192.4364	163.2242-235.7975	12.880	<0.05
**W M**	−6.6009	2.7414 ± 0.365	255.7486	159.3871-405.0692	750.4194	459.8467-2245.3156	26.381	<0.05
**C M**	−9.6475	4.4181 ± 1.524	152.6357	94.5358-262.7553	297.6718	202.8848-6675.4919	18.475	<0.05

W: wild, C: cultivated, EO: essential oil, T: total extract, PE: petroleum ether fraction, M: methanol fraction, SE: standard error, LC _50_ : lethal concentration to cause 50% mortality in population, LC _90_ : lethal concentration to cause 90% mortality in population, CI: confidence interval, χ^2^: heterogeneity about the regression line.

## Discussion

Many researchers have already studied larvicidal potentials of plant derived compounds, extracts and essential oils against various insects, with the aim of finding active phyto-chemicals to replace synthetic insecticides. High cost of various commercial insecticides beside their food and environmental safety concerns, toxicity problems and increasing resistance rates have made their utilization undesirable (25, 26). Anti-larval activity of essential oil from a wild grown *B. persicum* against *An. stephensi* and *Culex pipiens* has been previously reported with LC _50_ values of 27.72 and 20.61ppm respectively (27). According to the results of our study, the essential oil, methanol total extract and petroleum ether fraction of both wild and cultivated samples had significant larvicidal activity against late third and early fourth instar larvae of *An. stephensi*. The larvicidal potential of γ-terpinene, cuminaldehyde and ρ-cymene, main constituents of both wild and cultivated type *B. persicum* fruits, against various insect larvae has been previously proved in several experiments. γ-Terpinene has shown potent larvicidal activity with LC _50_ value of 29.21 ppm against *Anopheles anthropophagus* (28) and 30.7 and 29.8ppm against *Aedes aegypti* and *Aedes albopictus* respectively (29). Zahran and Abdelgaleil (30) documented toxicity of cumin aldehyde on *Culex pipiens* larvae, which was more stronger than other tested monoterpenes in that experiment, with LC _50_ values of 38.9 and 21.4ppm for 24 and 48h exposures respectively. Anti-larval potential of ρ-cymene, the other main constituent, towards *A. aegypti* and *Ae. albopticus* has also been demonstrated (LC _50_ = 19.2 and 46.7ppm) (29). Higher lethal effect of the petroleum ether fraction in comparison to the methanol fraction, suggests higher potency of non-polar components than polar phenolics towards *An. stephensi* larvae. LC _50_ value of 85.9933 and 62.7814ppm for petroleum ether fraction from wild and cultivated types makes it suitable choice for further studies to isolate the active principles. Anti-larval activity of efficient samples from cultivated type was comparable to those from wild grown, so it can be concluded that cultivation of *B. persicum* has not affected chemical constituents’ biosynthesis or concentration, which are responsible for larvicidal activity of the fruit.

## Conclusion

The extract and fractions from *B. persicum* fruits, ie, petroleum ether fraction and total extract, beside the essential oil, have shown significant larvicidal effects on *An. stephensi*, and can be a great candidate to develop an eco-friendly insecticide to combat malaria vector breeding. More precise investigation will require revealing phytochemical composition of extract. Since cultivated type showed comparable results as wild grown, cultivation of *B. persicum*, as a solution to preserve its wild resources, is highly recommended. There are several studies on larvicidal activities of different plants against malaria vectors in Iran (16, 31–46). We recommend formulation of plant extract which have the lowest LC _50_ for field evaluation.
